# Risk factors for atypical femur fractures with and without bisphosphonate treatment: a national nested case-control study

**DOI:** 10.1093/jbmrpl/ziag059

**Published:** 2026-04-03

**Authors:** Rasmus Mikiver, Rickard Sand, Hans Peter Bögl, Georg Zdolsek, Daphne Wezenberg, Karl Michäelsson, Johan Lyth, Jörg Schilcher

**Affiliations:** Department of Biomedical and Clinical Sciences, Linköping University, 581 83, Linköping, Sweden; Clinical Department of Orthopaedics in Linköping, Region Östergötland, 581 83, Linköping, Sweden; Department of Health, Medicine, and Caring Sciences, Linköping University, 581 83, Linköping, Sweden; Department of Biomedical and Clinical Sciences, Linköping University, 581 83, Linköping, Sweden; Clinical Department of Orthopaedics in Linköping, Region Östergötland, 581 83, Linköping, Sweden; Department of Biomedical and Clinical Sciences, Linköping University, 581 83, Linköping, Sweden; Clinical Department of Orthopedic Surgery in Gävle, Region Gävleborg, 801 87, Gävle, Sweden; Centre for Research and Development, Uppsala University, 801 87, Gävle, Sweden; Department of Biomedical and Clinical Sciences, Linköping University, 581 83, Linköping, Sweden; Clinical Department of Orthopaedics in Linköping, Region Östergötland, 581 83, Linköping, Sweden; Department of Biomedical and Clinical Sciences, Linköping University, 581 83, Linköping, Sweden; Clinical Department of Orthopaedics in Linköping, Region Östergötland, 581 83, Linköping, Sweden; Department of Surgical Sciences-Medical Epidemiology, Uppsala University, 751 85, Uppsala, Sweden; Department of Health, Medicine, and Caring Sciences, Linköping University, 581 83, Linköping, Sweden; Department of Biomedical and Clinical Sciences, Linköping University, 581 83, Linköping, Sweden; Clinical Department of Orthopaedics in Linköping, Region Östergötland, 581 83, Linköping, Sweden; Wallenberg Center for Molecular Medicine, Linköping University, 581 83, Linköping, Sweden

**Keywords:** atypical femur fracture, bisphosphonates, stress fractures, femoral geometry, national registry study, risk factors, osteoporosis treatment

## Abstract

Atypical femur fractures (AFF) are rare stress fractures linked to long-term bisphosphonate (BP) use. However, since some patients develop AFF without BP exposure (BP-naïve), additional mechanisms have been proposed. In this study, we tested medication use, clinical characteristics, and femoral geometry. We started with a hypothesis that BP-naïve patients would have additional risk factors in these areas compared with BP-users. We included 172 patients aged ≥55 yr who sustained AFF in Sweden between 2008 and 2010. Among them, 134 had used BPs prior to the fracture and 38 had not. All fractures met the 2014 ASBMR major criteria for AFF. Data on medication use, comorbidities, and demographics were extracted from national registers, and radiographs were reviewed to assess femoral geometry. All statistical models were adjusted for sex and age. We found that among BP-naïve patients, male sex was more common (21.1%) than among BP-users (3.0%). Additionally, BP-naïve patients were less likely to use calcium supplementation (23.7% vs 90.3%, OR 0.04, 95% CI 0.01-0.10), corticosteroids (13.2% vs 35.8%, OR 0.18, 95% CI 0.05-0.52), proton pump inhibitors (23.7% vs 41.8%, OR 0.36, 95% CI 0.14-0.85), and beta-blockers (28.9% vs 47.0%, OR 0.43, 95% CI 0.18-0.97). Moreover, BP-naïve patients had fewer diagnosed bone disorders, including osteoporosis (5.3% vs 34.3%, OR 0.10, 95% CI 0.02-0.38). Bisphosphonate-naïve patients did have a higher lateral-to-medial cortical thickness ratio (1.00 vs 0.90, *p* < .01); however, other geometric differences were minor and did not support the hypothesis. The absence of identifiable mechanisms for stress fracture formation suggests that future research should test factors not captured in registry data or femoral geometry. Possibly, physical activity levels or subtle bone metabolic conditions may be contributing to the pathogenesis of AFF, particularly in BP-naïve individuals.

## Graphical Abstract

**Graphical Abstract:**
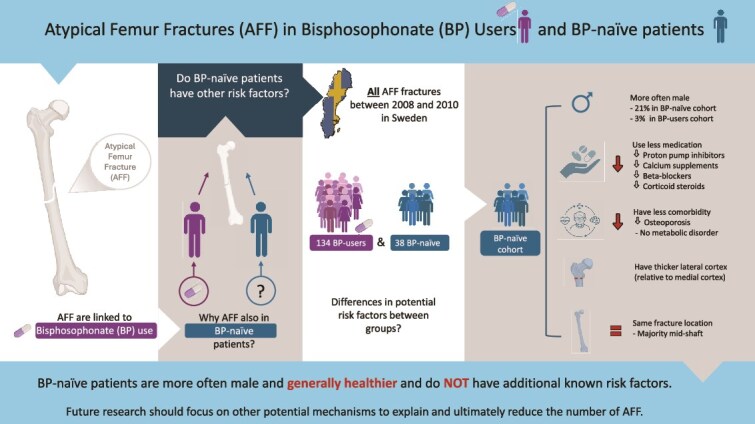

## Introduction

A rare but serious bone condition in older adults is atypical femur fracture (AFF). Radiographically, these AFFs in older patients resemble stress fractures that can be found in especially younger female athletes (fatigue type stress fractures), or as a result of bone metabolic conditions such as osteogenesis imperfecta and hypophosphatasia (insufficiency type stress fracture); these fractures are characterized by transverse fracture lines and focal cortical thickening (the healing reaction).^1^^,^^2^ However, whereas stress fractures in younger athletes are underlined by repetitive high-load mechanical activity, such activity is generally absent in older adults with AFFs. Instead, among older adults, AFFs are typically linked with the use of bisphosphonate (BP) treatment for osteoporosis. Bisphosphonates are widely used in elderly populations to reduce the risk of osteoporosis-related fragility fractures, and for this they are considered effective.^3^ Nevertheless, BP treatment over long time periods has also been clearly associated with AFFs.^4–8^ According to estimates in some studies, the long-term use of BP compared to non-use can, depending on the duration of use, increase the risk of AFFs by as much as 40 times.^5^^,^^6^ Further confirming the association, after BP treatment is discontinued, higher risks of AFF rapidly diminish.^4^^,^^6^

In older patients using BPs who present with AFFs, there is not normally the kind of activity characteristic of young athletes. Instead, AFFs are thought to develop from normal physiological loading, but on bone that has been structurally compromised by insufficiency-type mechanisms.^9^ Specifically, several pathophysiological mechanisms have been proposed to explain AFFs in patients with long-term BP treatment (BP-users): (1) altered bone matrix composition impairing mechanical properties^10^; (2) suppression of targeted remodeling, leading to microdamage accumulation^11^; and (3) pathological femoral geometry that has predisposed individuals to increased local stress concentrations.^12^^,^^13^ Collectively, these proposed mechanisms would suggest that AFFs in BP-users are primarily associated with some combination of impaired bone quality and unfavorable geometry.

Despite the clear plausibility of these mechanisms, and despite the clear association between AFFs and BPs, uncertainty remains about the causes of AFFs. Notably, among older adults, there are some patients who present with AFF without having any documented exposure to BP treatment (patients who are “BP-naïve”). Further complicating matters, there is a wide variation in the reported incidence of these BP-naïve cases. Some studies report the incidence of AFF cases without documented BP exposure to have been as low as 2.6%, but other studies report it to have been as high as 72.0%.^4^^,^^6^^,^^14–17^ This wide variation might be the result of differences in the duration and type of BP use, subjective differences in radiographic criteria for adjudication of AFF, and differences in the populations studied.^18^ However, some degree of this variation may also suggest that some cases of AFF are associated with an interplay of multiple other factors beyond BP.

In the literature, proposals have been made for several possible contributors to AFF other than BP. The primary contributors that have been proposed include certain medications, metabolic and inflammatory comorbidities, genetics, and anatomical variations in hip and femoral geometry, including those correlated with certain populations or ethnicities. All of these possible contributors, it is thought, could predispose patients to focal mechanical overload.^12^^,^^19–21^ However, actually testing these possible contributors to AFF has been difficult. Atypical femur fractures are rare, particularly in BP-naïve individuals, making them hard to study in sufficiently powered cohorts. An additional challenge is confirming true BP exposure status; most registry-based studies rely on prescription data as a proxy, which can be incomplete and thus underestimate actual treatment.^6^^,^^15–17^^,^^22^ While some studies have explored AFFs in patients without BP treatment,^14^^,^^23^^,^^24^ small sample sizes seem likely to have been a hindrance, and they have not been able to identify clear or consistent risk factors distinguishing these patients from BP-users. A step in the right direction, at least, would be to study larger sample sizes with comprehensive radiographic adjudication.

This study aimed to use one of the largest existing cohorts of radiographically confirmed AFF patients (*N* = 172) to examine risk factors both for patients with documented BP treatment (BP-users) and for patients with no documented use of BP (BP-naïve). Based on a literature review of the possible non-BP contributors to AFF that have been proposed, we decided to use this relatively large cohort to test the following hypothesis: BP-naïve patients would display distinct patterns of drug exposure, comorbidities, or femoral geometry that could indicate alternative stress fracture mechanisms compared to the BP-driven development of stress fracture in BP-users. Whether or not such risk factors could be identified might, in turn, be useful when considering ways that osteoporosis treatment could be tailored to minimize the risk of AFF while still preventing fragility fractures.

## Materials and methods

### Study population

**Figure 1:**
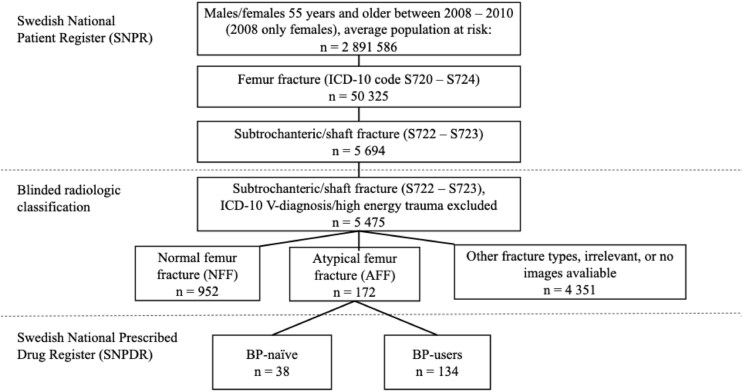
Identification of 172 atypical femur fractures (AFFs) through radiographic review of 5475 subtrochanteric/diaphyseal fractures; 38 were BP-naïve and 134 BP-users.^4^

The patients included in this nationwide nested case-control study were previously identified based on the Swedish National Patient Register (SNPR) (Figure 1).^4^^,^^5^ The cohort comprised individuals aged 55 yrs and older who were hospitalized with a femur fracture (ICD-10 codes S720-S724) between January 1, 2008 and December 31, 2010. For the year 2008, only females were included. Information on race or ethnicity is not recorded in Swedish national health registers and could therefore not be included as a covariate in the present study. The Swedish population is predominantly of Northern European ancestry.^25^

Over this period, 38 747 female and 11 578 male patients with a femur fracture were identified. Among them, 5694 patients experienced a femoral subtrochanteric or shaft fracture (diagnosis codes S722 or S723, ICD-10). For patients with admissions for multiple femur fractures during the study period, whether ipsilateral or contralateral, only their initial fracture event was considered. Patients with injuries and ICD-10 V-diagnosis codes associated with motor vehicle accidents, as well as those with falls from heights greater than standing, were excluded.

The remaining 5475 cases were subject to radiographic adjudication. Digital radiographs from 76 hospitals were reviewed by trained adjudicators blinded to patient characteristics. A total of 172 patients met the 2014 American Society for Bone and Mineral Research (ASBMR) major criteria for AFF (Figure 1).^2^ The ASBMR major criteria—transverse fracture line and focal cortical thickening—were compulsory in our case definition of AFF to accurately reflect the radiographic features of stress fractures.

Of the 172 AFF patients, 134 had claimed ≥1 prescription of BPs before the fracture, as indicated by data from the Swedish National Prescribed Drug Register (SNPDR) (BP-users). The remaining 38 patients had no documented use of oral BPs from July 2005 until the date of fracture (BP-naïve). A manual review of medical records was conducted for 36 of these 38 patients, covering at least 3 yr prior to fracture, and confirmed no use of antiresorptive therapy (unpublished data).

### Registry information

For all patients, data were linked across multiple national health registries in Sweden using the unique personal identification numbers assigned to all Swedish residents. Information on comorbidities was obtained from the SNPR, while data on medication use were extracted from the SNPDR. The inclusion period for registry data was defined as follows: inpatient diagnoses from 1998 onward, outpatient diagnoses from 2001 onward, and prescribed drug data from July 2005 (the start of the SNPDR). All data were collected up to the date of the fracture.

### Coding of diseases and drug treatments

A literature review was conducted to identify medications, comorbidities, and pathological variations in femoral geometry potentially associated with the development of stress fractures and AFF. Based on this review, relevant diagnostic codes from the International Classification of Diseases, 10th Revision (ICD-10), and Anatomical Therapeutic Chemical (ATC) classification system were identified and compiled into approximately 70 drug categories and 20 disease groups, which formed the basis for the parameters used in the statistical analyses.

### Assessment of femoral geometry

**Figure 2:**
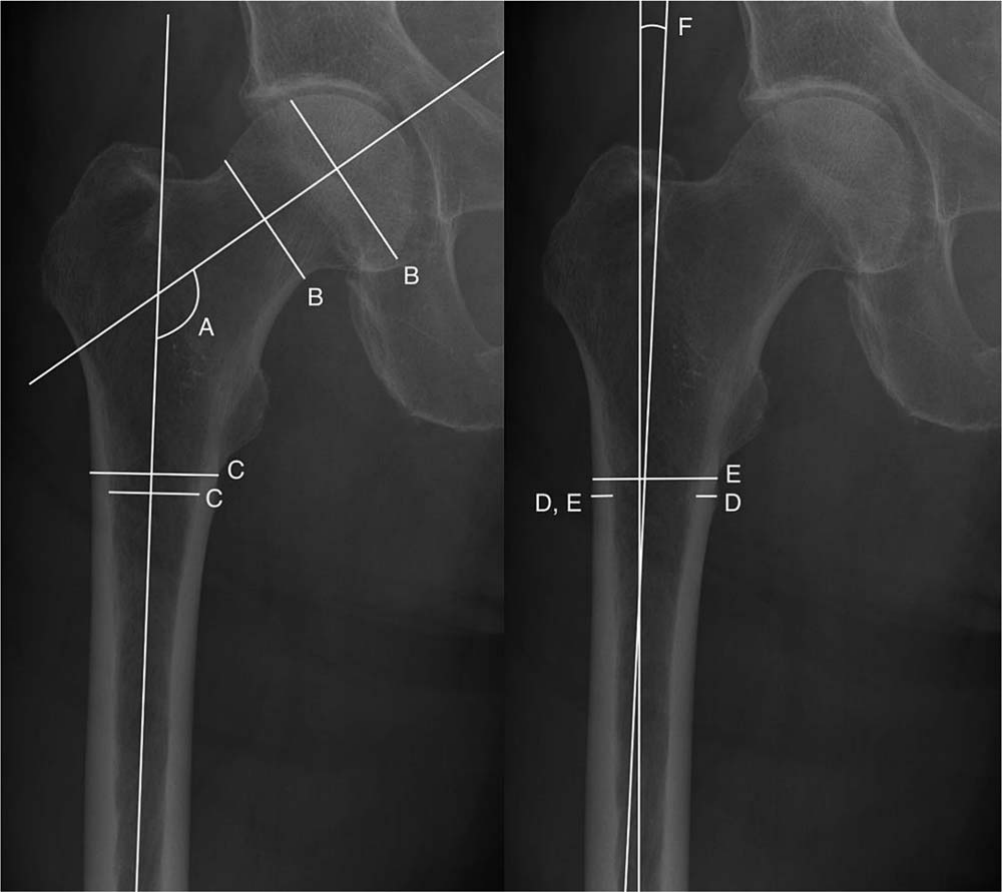
Assessment of femoral geometry on a 2-dimensional anteroposterior radiograph of a femur on the contralateral side relative to the fracture. A = Femoral neck-shaft angle. B = Head-neck offset ratio. C = Total cortical thickness index (CTi). D = Lateral to medial cortical thickness ratio. E = Lateral cortical thickness index (LCTi). F = Lateral femoral bowing.

Radiographic measurements of femoral geometry were performed (R.S.) using 2-dimensional radiographs. Image selection followed a predefined hierarchy based on availability: (1) contralateral femur, (2) ipsilateral femur prior to fracture (including incomplete AFFs), and (3) ipsilateral femur after fracture. All measurements were conducted using the Sectra IDS7 Picture Archiving and Communication System (PACS) software (Sectra AB). Radiographs from all 172 AFF patients were assessed with the investigator blinded to all background information. Structural parameters were selected based on the literature review and included: femoral neck-shaft angle, femoral head-neck offset ratio, total cortical thickness index (CTi), lateral-to-medial cortical thickness ratio, lateral cortical thickness index (LCTi), and lateral femoral bowing^24^^,^^26–28^ (Figure 2 and Appendix). All measurements were performed according to a standardized protocol.

In addition to geometric measurements, the anatomical location of the fracture along the femoral shaft was assessed using radiographs. We applied a previously described method to distinguish between subtrochanteric and shaft fractures based on fracture location.^29^ Subtrochanteric fractures were located within 80 mm distal of the lesser trochanter; all other fractures were classified as shaft fractures. When feasible, the total femur length was also assessed by measuring the distance from the greater trochanter to the fracture line and from the fracture line to the distal femoral condyles. All measurements were conducted without reference scale calibration.

### Statistics

The crude incidence per 100 000 person-years of AFF among females and males aged 55 and older, as well as among those with femur fractures, was calculated for the years 2009-2010. Differences in mean age between the 2 groups were analyzed with a *t*-test. To examine whether BP use was associated with differences in comorbidities and drug treatments among patients with AFF, we conducted univariable and multivariable logistic regression analyses among patients with AFF with and without BP exposure, in a nested case-control study. In these models, the independent variables were the presence or absence of specific comorbidities or medication use, and the dependent variable was BP treatment status (BP-users vs BP-naïve), with the BP-naïve group used as the reference. Estimates were adjusted for sex and age. The proportion of patients with BP treatment dependent on age was used to create a spike histogram. Based on the age distribution, we accounted for potential non-linear effects of age in the logistic regression models using restricted cubic splines with 3 knots.^30^

Results are reported as frequencies and odds ratios (OR) with 95% CI. Forest plots are used to display adjusted odds ratios and 95% CI from the multivariable models, with the BP-naïve group as the reference. Age was presented using the likelihood of BP-naïve patients dichotomized at a cut-off at 70 yr, inspired by Kilgour et al.^30^ For parameters of femoral geometry, descriptive statistics were calculated, and age- and sex-adjusted mean differences were analyzed using linear regression. Missing data was excluded through pairwise deletion. All statistical tests were 2-sided, and *p*-values <.05 were considered statistically significant. Analyses were performed using R Statistical Software (version 4.5.1; R Core Team, 2020).

### Ethics

Ethical approval was applied for and granted by the regional ethics board in Linköping through applications (2011/358-31) and (2022-04383-02).

## Results

### Incidence

Atypical femur fractures were rare in the general population. For the years 2009-2010, the incidence was 3.5 for females and 0.4 for males per 100 000 person-years among people aged 55 yr and older. The incidence of all other femur fractures (excluding AFF) was 16.0 per 100 000 person-years for females and 6.5 for males. The incidence of AFFs in BP-naïve individuals was 0.5 per 100 000 person-years (females 0.7, males 0.3) and for BP-users 45.4 (females 49.9, males 15.5).

**Figure 3:**
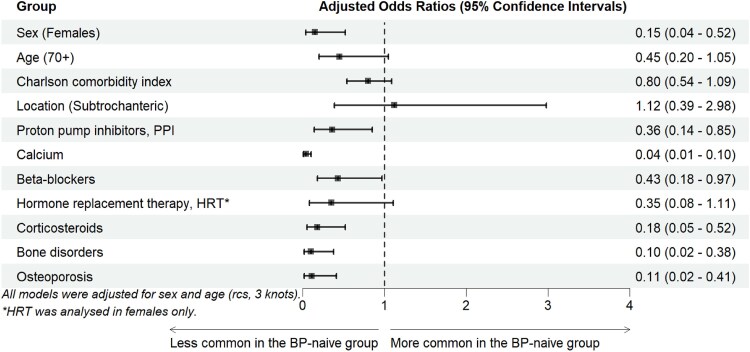
Sex- and age-adjusted models of factors associated with BP treatment in patients with atypical femur fractures (AFFs).

Among all femur fractures (S72.0-S72.4), between 2008 and 2010, AFFs accounted for 0.3%. Within the subgroup of subtrochanteric and femoral shaft fractures (S72.2 + S72.3), AFFs represented 15.3% after exclusion of specific fracture types (NFF and AFF, Figure 1).

### Patient characteristics

**Figure 4:**
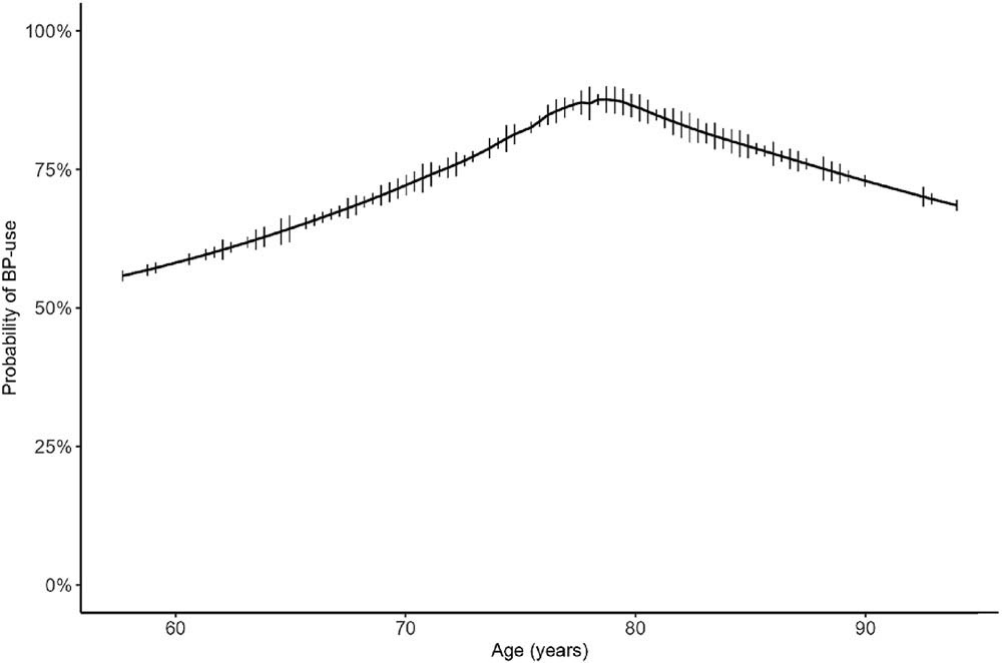
Probability of BP use by age.

The mean age at fracture was 74.8 (SD 9.3) in BP-naïve patients compared with 77.3 (SD 7.8) in BP-users (*p* = .14) (Table 1). Male sex was more common among BP-naïve patients (21.1%) than among BP-users (3.0%), and age-adjusted logistic regression confirmed lower odds for females (OR 0.15, 95% CI 0.04-0.52) (Figure 3). Using a 70-yr cutoff, the proportion below that threshold was larger in BP-naïve patients than in the BP-group but did not reach statistical significance (sex adjusted OR 0.45, 95% CI 0.20-1.05, *p* = .06; Figure 3). The proportion of patients with BP treatment increased up to the age of roughly 80 yr and decreased steadily thereafter (Figure 4).

**Table 1:** Patient characteristics of BP-users and BP-naïve patients with atypical femur fractures (AFFs).

	**AFF group**	
	**BP-naïve**	**BP-users**
	**(*n* = 38)**	**(*n* = 134)**
**Background information**		
**Age, mean (SD)**	74.8 (9.3)	77.3 (7.8)
**Sex, *n* (%)**		
**Male**	8 (21.1)	4 (3.0)
**Females**	30 (78.9)	130 (97.0)
**Charlson comorbidity index, mean (SD)**	0.55 (0.92)	0.76 (1.46)
**Location, *n* (%)**		
**Shaft**	28 (73.7)	111 (83.5)
**Subtrochanteric**	10 (26.3)	22 (16.5)
**Missing (%)**	-	1 (0.01)
**Selected drugs and comorbidities, *n* (%)**		
**Proton pump inhibitors** ^**a**^	9 (23.7)	56 (41.8)
**Calcium** ^**b**^	9 (23.7)	121 (90.3)
**Beta-blockers** ^**c**^	11 (28.9)	63 (47.0)
**Hormone replacement therapy** ^**d**^	3 (7.9)	31 (23.1)
**Corticosteroids** ^**e**^	5 (13.2)	48 (35.8)
**Bone disorders** ^**f,g**^	2 (5.3)	46 (34.3)
**Osteoporosis**^**f**^	2 (5.3)	43 (32.1)
**Other bone disorders**^**g**^	-	4 (3.0)

ATC codes: ^a^A02BC, ^b^A12A, ^c^C07, ^d^G03CA03, ^e^H02AB. ICD-10 codes: ^f^M80, M81, ^g^M85.

### Comorbidities and medications

Figure 3 shows comorbidities and medications that differed significantly between BP-naïve and BP-users prior to adjustment, including the background characteristics sex, age, and the Charlson comorbidity index. After adjustment for sex and age (restricted cubic splines), several factors were significantly less common in the BP-naïve group: the use of proton pump inhibitors (PPIs) (23.7% vs 41.8%, OR 0.36, 95% CI 0.14-0.85), calcium supplementation (23.7% vs 90.3%, OR 0.04, 95% CI 0.01-0.10), beta-blockers (28.9% vs 47.0%, OR 0.43, 95% CI 0.18-0.97), and corticosteroid use (13.2% vs 35.8%, OR 0.18, 95% CI 0.05-0.52). Additionally, bone metabolic disorders, including osteoporosis, were markedly less prevalent among BP-naïve patients (5.3% vs 34.3%, OR 0.10, 95% CI 0.02-0.38). We did not identify any diagnostic codes linked to bone-metabolic conditions (other than osteoporosis) or genetic bone diseases among BP-naïve patients.

### Measurements of femoral geometry

Table 2 displays the unadjusted mean values and sex and age (continuous) adjusted mean differences for the measurements of femoral geometry. Patients in the BP-naïve group had a larger lateral-to-medial cortical thickness ratio: 1.00 vs 0.90, *p* < .01.

**Table 2:** Comparison of femoral geometry in BP-users and BP-naïve patients.

	**AFF group**		**Adjusted mean difference (95% CI)**	***p*-value**
	**BP-naïve**	**BP-users**		
	(*n* = 38)	(*n* = 134)		
**Femoral neck-shaft angle (degrees)**				
**Mean (SD)**	128 (8.02)	128 (6.78)	0.46 (−2.33; 3.24)	.75
**Median (min, max)**	128 (115, 142)	128 (110, 152)		
**Missing (%)**	2 (5.3)	8 (6.0)		
**Femoral head-neck offset ratio**				
**Mean (SD)**	0.71 (0.03)	0.71 (0.04)	0.00 (−0.01; 0.02)	.65
**Median (min, max)**	0.70 (0.66, 0.81)	0.71 (0.60, 0.86)		
**Missing (%)**	1 (2.6)	5 (3.7)		
**Cortical thickness index (CTi)** ^a^				
**Mean (SD)**	0.33 (0.09)	0.33 (0.07)	0.00 (−0.03; 0.03)	.92
**Median (min, max)**	0.30 (0.23, 0.54)	0.32 (0.14, 0.51)		
**Missing (%)**	3 (7.9)	8 (6.0)		
**Lateral to medial cortical thickness ratio** ^a^				
**Mean (SD)**	1.00 (0.27)	0.90 (0.18)	−0.11 (−0.19; −0.03)	<.01
**Median (min, max)**	0.96 (0.59, 1.79)	0.89 (0.50, 1.52)		
**Missing (%)**	3 (7.9)	8 (6.0)		
**Lateral cortical thickness index (LCTi)** ^a^				
**Mean (SD)**	0.16 (0.04)	0.15 (0.03)	−0.01 (−0.02; 0.01)	.28
**Median (min, max)**	0.15 (0.11, 0.29)	0.15 (0.07, 0.25)		
**Missing (%)**	3 (7.9)	8 (6.0)		
**Lateral femoral bowing (degrees)**				
**Mean (SD)**	3.49 (3.10)	3.32 (3.17)	−0.01 (−1.22; 1.20)	.98
**Median (min, max)**	2.30 (0.10, 13.9)	2.50 (0.00, 19.7)		
**Missing (%)**	3 (7.9)	9 (6.7)		

a LT is measured at the lesser trochanter level.

All pairwise comparisons adjusted for sex and age.

Abbreviation: 95% CI, 95% confidence interval.

### Fracture location

The proportion of fractures in the subtrochanteric area was 26.3% (*n* = 10) in the BP-naïve group compared to 16.5% (*n* = 22) in the BP-users, age- and sex-adjusted *p* = .82.

## Discussion

Our hypothesis in this study was based on proposals in the literature for several possible contributors to AFF other than BP. Of these, the primary contributors we considered were certain medications, comorbidities, and anatomical variations in hip and femoral geometry that could predispose to stress fracture formation in the femoral shaft.

In our extensive comparison of potential risk factors for AFF obtained from registries and radiographic analyses, we found no support for the hypothesis that BP-naïve patients would have a higher likelihood of drug exposures, comorbidities, and biomechanical deviations in femoral geometry. As would be expected from the existing literature, our study did confirm that BP-naïve patients were more likely to be male.^14^^,^^20^^,^^23^ Contrary to the hypothesis about proposed contributors, though, we found that BP-naïve patients actually exhibited fewer medications and bone disorders.

Regarding femoral geometry, we found only one statistically significant difference, a thicker lateral cortex relative to the medial at the level of the lesser trochanter in the BP-naïve group. Finally, we noted that most of the AFFs in our study were found in the midshaft area, which complicates previous reporting on fracture location. These findings raise further questions about the relationship between AFF and anatomy.

The lack of support for the hypothesis about drug exposures, comorbidities, and femoral geometry led us to consider other aspects of AFF in BP-users vs BP-näive patients that may warrant further discussion and investigation, which we mention below.

### Supplements, drugs, and comorbidities

Compared to BP-users, the BP-naïve group had a lower prevalence of nearly all types of medications and comorbidities. Among patients who are prescribed BPs, calcium is commonly co-prescribed to support bone health, and corticosteroids are often combined with BPs to mitigate bone loss.^31^ In addition, patients who are prescribed BPs often have a diagnosis of a bone disorder; for example, in our study the most common such disorder among BP-users was osteoporosis. As should be expected, our BP-naïve group had, by contrast, levels of calcium supplementation, corticosteroid treatment, and bone disorders that were lower than BP-users.^31^

Another medication frequently prescribed to BP-users is PPIs, used to address gastroesophageal symptoms, a common side effect of oral BP treatment.^32^ Some studies have suggested that PPIs themselves could increase the risk of fragility fractures, by way of disruptions to the gastric absorption of nutrients and minerals, such as calcium.^33^ Whether this link affects the mechanism of AFF is unclear. In our study, the BP-naïve group in the sex- and age-adjusted models exhibited a lower use of PPIs, again as might be expected. Confirming the existing literature, our study suggests that in BP-naïve patients,^32^^,^^33^ PPI use does not appear as a strong driving factor for AFF.^14^^,^^23^^,^^24^^,^^34^

Finally, another medication of interest is beta-blockers, often used for rate-control therapy for atrial fibrillation. Beta-blockers were included as a pre-specified variable because of previous reports suggesting potential effects on bone metabolism and fracture risk.^35–39^ In our study, BP-naïve patients also had lower rates of beta-blocker prescriptions compared with BP-users. This difference is most plausibly explained by differences in comorbidity profiles and by the known association between BP therapy and atrial fibrillation, for which beta-blockers are often prescribed.^40^

### Femoral geometry

In trying to assess possible contributors to AFF, 2 measures of femoral geometry seem particularly relevant: (1) lateral cortical thickness index (LCTi) and (2) lesser trochanter–lateral to medial cortical thickness ratio. Both measures seem to argue for a mechanical imbalance that favors stress concentration laterally, which is a key feature in the pathogenesis of AFF. The first measure—LCTi—was used in a recent study of AFF patients, in this case of Asian ethnicity, to compare BP-users and BP-naïve patients; the study found that BP-users had a significantly greater LCTi than BP-naïve patients.^27^ The second measure—lesser trochanter–lateral to medial cortical thickness ratio—had, to our knowledge, not previously been used in a comparison of BP-user and BP-naïve AFF patients. We tested this measure in our study and found a divergent result: significantly greater thickness in BP-naïve patients than in BP-users (Table 2). The contradictory findings between these 2 studies might be related to population-based differences in the areas of the femur where AFFs tend to occur. Among Asian populations, AFFs in BP-users appear to be more commonly located in the subtrochanteric region, whereas in Caucasians the most common location is in the femoral diaphysis.^29^

Otherwise, within our study, femoral geometry differences between the BP-user and BP-naïve groups were moderate and did not reach statistical significance. This included a lack of significant differences in lateral femoral bowing and femoral neck-shaft angle (varus geometry). Although these factors have been previously linked to AFF, the link has been inconsistent, and it has been demonstrated mainly in Asian cohorts,^15^^,^^19^^,^^22^^,^^41^ where again, particular population-based effects may be in play. In Asian cohorts, bowing in the femoral-shaft curvature correlates with fracture location, with greater bowing associated with more distal fractures, and this is independent of BP use.^24^ So, overall, femoral geometry as a possible contributor to AFF in BP-users and BP-naïve patients alike may be predominantly a matter of general population-related tendencies.

### Fracture location

Another approach to testing possible contributors to AFF in BP-users vs BP-naïve patients has been to look at the area of the femur affected. Among fractures that are categorized as AFFs, it has been proposed that there are “typical” AFFs, but also other subtypes, with varying fracture locations. In the approach proposed by Oh et al., “typical” AFFs are found primarily in BP-users, are linked to suppressed bone turnover, and are located primarily in the subtrochanteric area.^42^ Unlike these “typical” AFFs, Oh et al. then go on to suggest, there is also a subtype of AFFs that are more commonly found in bowed femurs, are associated with preserved bone remodeling, and are located primarily in the midshaft. According to Oh et al., subtrochanteric AFFs could reflect insufficiency-type mechanisms related to impaired remodeling under normal loading, while midshaft AFFs may represent fatigue-type stress fractures resulting from mechanical overload on a bowed shaft, independent of antiresorptive therapy.^42^ This theory is supported by findings from Thailand, where a higher proportion of subtrochanteric AFFs in BP-users has been reported compared to BP-naïve patients.^27^ However, in our study, all AFFs were predominantly located in the midshaft region of the femur, regardless of BP exposure. This divergence, as well, may be partly explained by population-related differences in femoral geometry that increase local mechanical stress, such as greater bowing and shaft width among Asian populations compared with Caucasians.^43^^,^^44^ Thus, beyond general population-related tendencies, the significance of fracture location for deciphering the causes and types of AFF remains an open question.

### Age

Given the challenges of studying AFFs and the lack of clear findings so far about the possible roles of drug exposures, comorbidities, femoral geometry, and fracture location in assessing AFFs in BP-naïve patients as well as BP-users, other areas of investigation might also be worth considering. One such area is age. Among older adults, age generally correlates with differences in physical activity, musculoskeletal function, and general health status.^30^ Older adults typically exhibit declines in physical function, gait stability, and exercise tolerance. By contrast, younger individuals tend to maintain higher activity levels, mobility, and skeletal loading. These functional differences may influence AFF pathophysiology.^20^^,^^30^^,^^45^ In older, less active adults, insufficiency-type fractures, resulting from normal loading on weakened bone, may predominate. However, in younger, active individuals, fatigue-type stress fractures, caused by repetitive loading of otherwise healthy bone, may be more common. A consistent trend in the literature suggests that compared with other femoral fractures, AFF patients do tend to be younger, and indeed, within AFF patients as a whole, compared with BP-users, BP-naïve patients tend to be the younger group, which makes it more likely that there is an association in these patients between AFF and higher activity levels and skeletal loading.^20^^,^^23^^,^^45^

That being said, in our study, with age set as a continuous variable, we did not observe statistically significant age differences between the groups. This could be related to limitations on statistical power caused by a relatively small sample size, even though in comparison to other AFF cohorts, the cohort in our study had the advantage of being comparatively large.

There is another possibility, though, which is that modeling age as a continuous covariate may obscure threshold-dependent effects across a biologically heterogeneous population. Instead, a better approach could be to categorize patients by age, in order to better reflect mechanistic distinctions that align with functional changes at specific age thresholds. To be sure, this approach also has possible limitations; for example, relying on chronological age may introduce bias, because it does not equate to biological age, which cannot be assessed in registry data. Nevertheless, we decided to test this approach by conducting an additional exploratory analysis. We used a 70-yr threshold inspired by thresholds from Kilgour et al.,^30^ to better capture variation based on age thresholds. With our 70-yr cutoff, the proportion below that threshold was indeed larger in BP-naïve patients than in the BP-user group; however, the difference did not reach statistical significance. Nonetheless, we would recommend that future research consider the assessment of such effects.

However, an even better proxy for biological age is likely to be physical activity. To our knowledge, only one study has examined this. Adams et al. (2025) assessed whether a higher risk of AFF in patients using BPs was related to weekly exercise levels.^44^ That study considered ethnic differences in a cohort of 183 610 women. Adams et al. did find significant variation in exercise levels. In that study, though, these differences in exercise levels, as well as differences in AFF risk, correlated with ethnicity, and adjusting for exercise activity did not reduce ethnicity-correlated AFF risk. Again, this is likely due to strong population-related tendencies—in their study, for example, the risk of AFF for Asian women was five times higher than for other ethnicities. The Adams et al. study does provide a model, though, for collecting data on exercise levels as a more informative measure than just chronological age when examining possible scenarios around BP-user and BP-naïve AFF.

Together with other factors, such as genetic predisposition, these factors seem likely to be particularly relevant, since the fatigue-type stress mechanism could be making BP-users more susceptible to AFF while also contributing to a version of AFF in BP-naïve.^42^^,^^46^ Going forward, a key to further investigation could be to find ways to gather data, beyond register-based information, on physical activity and loading conditions,^47^ muscle strength, behavioral and genetic factors.

### Strengths and limitations

A key strength of our study is the relatively large number of AFF cases included, particularly given the rarity of this condition in the general population. Previous studies investigating AFF, especially in BP-naïve patients, have often been limited by very small sample sizes or lacked rigorous radiographic adjudication.^14^^,^^23^ By combining nationwide registry data with systematic radiographic review, we were able to define 2 well-characterized cohorts, allowing for a detailed comparison of BP-users and BP-naïve patients with AFFs. The methodology applied here provides a valuable framework for future analyses of AFF risk factors in larger populations.

However, important limitations should be acknowledged. The number of AFF cases in the BP-naïve group was still relatively small, limiting statistical power to detect subtle differences even in our study; this challenge is inherent to AFF research, since most cases of AFF globally come to light in connection with aspects of BP use, whereas BP-naïve cases of AFF are more likely to remain unreported.^6^ As for the consistency of our femoral geometry measurements, they were performed following a standardized protocol, but they were conducted by a single investigator. Meanwhile, although blinding minimized bias, inter-observer variability was not assessed; however, in other studies it has been excellent.^27^^,^^48^ Finally, few patients had full-length femur radiographs, so measurements of lateral femoral bowing were based on proximal or distal segments only and in most cases, was assessed on post-injury films, due to limited imaging.

Perhaps the most significant and interesting limitation from our perspective, however, is the use of register-based data, which lacks key variables such as physical activity, muscle strength, and behavioral factors that may influence skeletal loading. Furthermore, the register allowed the investigation of many covariates. Despite multiple statistical comparisons, we abstained from applying adjustment methods. The rationale was that Bonferroni-type corrections address the universal null hypothesis rather than individual associations and may increase the risk of type II errors, thereby having limited applicability when assessing evidence about specific hypotheses.^49^

### Conclusions

The findings in our study are in line with some of the previous research but contradict others and support the potential importance of both insufficiency and fatigue-type mechanisms in AFF patients with and without BP treatment. Bisphosphonate users may be more likely to experience insufficiency-type stress fractures due to BP treatment, while those in the BP-naïve group, comprised of younger patients with fewer comorbidities and medications, may develop fatigue-type fractures due to mechanical overload on healthy bone tissue. To be sure, it is likely that both mechanisms play an important role in both groups, possibly in association with variations of genetic predisposition. This multifactorial pathogenesis emphasizes the need, when AFF is identified, to assess as full a range of key variables as possible that could have been risk factors contributing to AFF, beyond registry-based data. This would be especially useful in cases of AFF, where no BP treatment is implicated, since these cases remain under-reported and under-studied but could reveal unique insights helpful to managing and trying to reduce all incidences of AFFs.

Future research should incorporate high-quality objective measures of physical activity, radiographic evaluation and genetic analysis in ethnically divergent populations to help elucidate the complexity of mechanisms that contribute to stress fracture risk beyond the very strong and well-established effect of antiresorptive therapy.

## Supplementary Material

Supplemental_material_ziag059

## Data Availability

Restrictions apply to the availability of some, or all of the data generated or analyzed in this study due to patient confidentiality and because certain data were accessed under license from Swedish national health registers. The data include individual-level health and radiographic information and are therefore subject to GDPR, the Swedish Public Access to Information and Secrecy Act, and ethical approvals. Consequently, the data are not publicly available. Information about the conditions for limited access to de-identified data may be obtained from the corresponding author upon reasonable request, subject to approval by the relevant authorities.
