# Supplementary material for: Risk factors for atypical femur fractures with and without bisphosphonate treatment: a national nested case-control study
Source: JBMR Plus. 2026 Apr 3;10(6):ziag059. doi: 10.1093/jbmrpl/ziag059 (PMC13156492; doi:10.1093/jbmrpl/ziag059)

Appendix

Femoral geometry protocol

A) **Femoral neck-shaft angle** = The angle between two lines: one parallel to the longitudinal axis of the femur and one parallel to the axis of the femoral neck bisecting the center of the femoral head and the center of the femoral neck (12).
B) **Head-neck offset ratio** = Femoral neck diameter measured perpendicular to the femoral neck, divided by the femoral head diameter drawn through center of the femoral head (12,25).
C) **Total cortical thickness index (CTi)** = Total femoral diameter subtracted by the medullary canal width, divided by the total femoral diameter (42).
D) **Lateral to medial cortical thickness ratio** = Lateral cortical thickness divided by medial cortical thickness (42).
E) **Lateral cortical thickness index** (LCTi) = Lateral cortical thickness divided by the total femoral diameter (26).
F) **Lateral femoral bowing** = The angle between two lines: one parallel to the intramedullary canal at the most proximal portion of the femur and one parallel to the most distal portion of the femur visualized on the radiograph (27). When measured on the ipsilateral side after fracture, the proximal or the distal fragment was chosen depending on which could be best visualized on the radiograph and the location of the fracture along the femoral shaft.

Lateral cortical thickness = A straight line drawn from the most lateral part of the femur to the most lateral part of the intramedullary canal in the same vertical plane, just distal to the most distal part of the lesser trochanter visible on the radiograph.
Medial cortical thickness = A straight line drawn from the most medial part of the femur to the most medial part of the intramedullary canal in the same vertical plane, just distal to the most distal part of the lesser trochanter visible on the radiograph.
Total femoral diameter = A straight line drawn perpendicular to the longitudinal axis of the femur from the most lateral part of the femur to the most medial part of the femur, just distal to the lesser trochanter.
Medullary canal width = Total femoral diameter subtracted by the sum of the lateral cortical thickness and medial cortical thickness.

Figure S1: Images used to assess proximal femoral geometry (includes all geometric measurements except lateral femoral bowing).

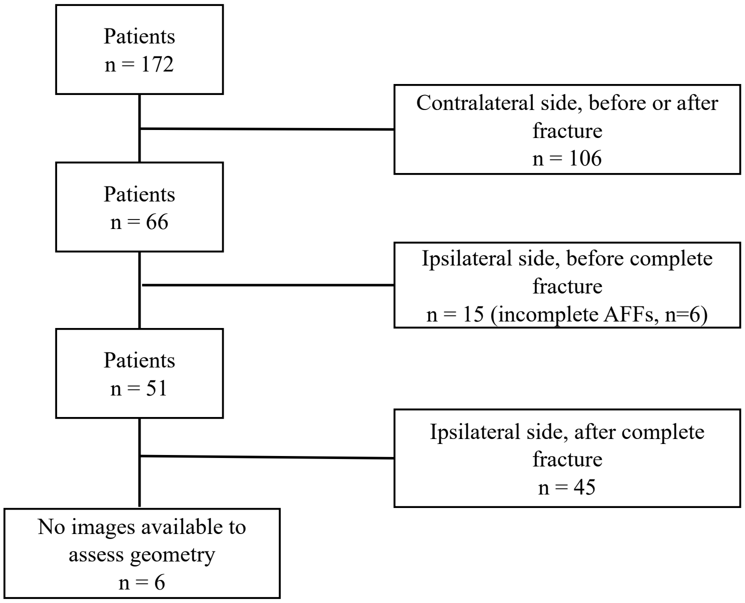

Figure S2: Images used to assess lateral femoral bowing.

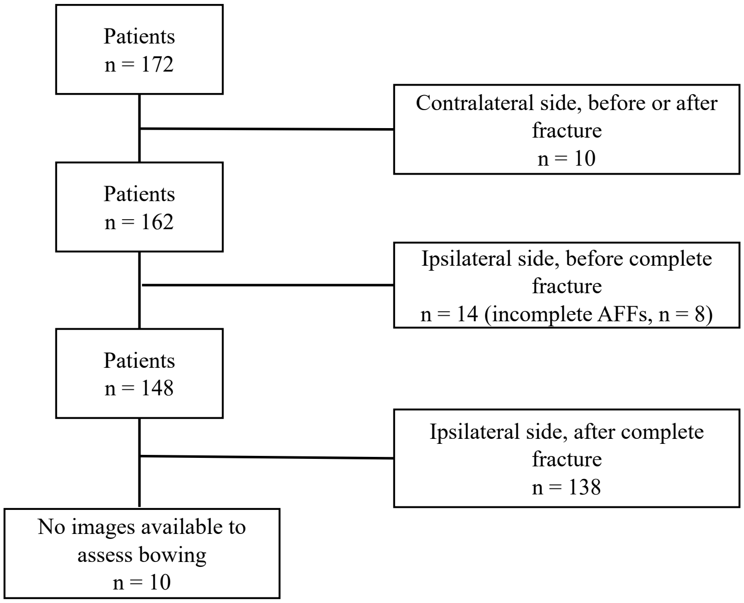

Supplement: Supplemental_material_ziag059 [file supplemental_material_ziag059.pdf]
